# Development of a subsurface LIBS sensor for in situ groundwater quality monitoring with applications in CO_2_ leak sensing in carbon sequestration

**DOI:** 10.1038/s41598-019-41025-3

**Published:** 2019-03-14

**Authors:** D. A. Hartzler, J. C. Jain, D. L. McIntyre

**Affiliations:** 1 National Energy Technology Laboratory, U.S. Department of Energy, Pittsburgh, PA 15236 USA; 2 National Energy Technology Laboratory, U.S. Department of Energy, Morgantown, WV 26505 USA; 3 Leidos Research Support Team, National Energy Technology Laboratory, Pittsburgh, PA 15236 USA

## Abstract

Sub-surface activity such as geologic carbon sequestration (GCS) has the potential to contaminate groundwater sources with dissolved metals originating from sub-surface brines or leaching of formation rock. Therefore, a Laser Induced Breakdown Spectroscopy (LIBS) based sensor is developed for sub-surface water quality monitoring. The sensor head is built using a low cost passively Q-switched (PQSW) laser and is fiber coupled to a pump laser and a gated spectrometer. The prototype sensor head was constructed using off the shelf components and a custom monolithic, PQSW laser and testing has verified that the fiber coupled design performs as desired. The system shows good calibration linearity for tested elements (Ca, Sr, and K), quick data collection times, and Limits of Detection (LODs) that are comparable to or better than those of table top, actively Q-switched systems. The fiber coupled design gives the ability to separate the PQSW LIBS excitation laser from the pump source and spectrometer, allowing these expensive and fragile components to remain at the surface while only the low-cost, all optical sensor head needs to be exposed to the hostile downhole environment.

## Introduction

Geologic carbon storage (GCS) involves the injection of carbon dioxide (CO_2_) into deep geological formations (e.g. saline aquifers, unmineable coal seams, and depleted oil and gas reservoirs) where it will be trapped permanently^1^. In fact, the injection of CO_2_ into depleted natural gas and oil reservoirs or unmineable coal beds has potential to displace trapped hydrocarbons resulting in enhancement of oil and gas recovery from these reservoirs^2,3^. Generally, saline formations are preferred for GCS because of their world-wide existence and large storage capacity, which make them a viable long-term storage solution. Although GCS appears to be a feasible pathway to reduce harmful CO_2_ emissions to the atmosphere, there are risks associated with the process if CO_2_ leakage were to occur. One of the potential risks associated with CO_2_ leakage is pollution of groundwater supplies by contaminants originating from acid leaching of injection formations and/or migration of formation fluids (e.g. hypersaline brines) into shallow groundwater aquifers^4,5^. Therefore, the detection of entrained contaminants that migrate into shallow groundwater aquifers can be used both to assess storage permanence and to evaluate the impacts of CO_2_ leakage on water resources. Moreover, monitoring of groundwater for elevated levels of a variety of dissolved metals (e.g., Na, Ca, Li, K, and Sr) could potentially provide an early detection of CO_2_ leakage.

Currently, a number of technologies such as inductively couple plasma mass spectrometry (ICP-MS) and inductively coupled plasma optical emission spectroscopy (ICP-OES) exist for the measurement of water quality^6^. These are mostly laboratory-based techniques and are not amendable to field measurements and analysis in a harsh-environment. These techniques may also suffer from interferences during analysis of highly saline and complex matrix water samples^7–10^ and could never be configured for an *in situ* downhole measurement. In general, the lab-based analytical techniques require samples to be retrieved and transported to a laboratory for preparation and analysis that is not only a laborious process but could potentially alter the sample chemistry and compromise the sample integrity^11^. While there are existing tools for downhole elemental analysis such as x-ray fluorescence (XRF), these tools require expensive components to be placed downhole. Moreover, XRF is not suited to measure light elements^12,13^.

Laser Induced Breakdown Spectroscopy (LIBS) is uniquely suited for rapid, *in situ* detection and quantification of dissolved metals in groundwater. LIBS is a rapidly advancing spectroscopic technique, which offers several analytical advantages including its capability for field deployment and real time analysis. The flexibility of probe design and use of fiber optics make it a suitable technique for measurements in harsh subsurface conditions and in hard to reach places. Previous work in our and other laboratories has shown that underwater LIBS can successfully be used for the elemental analysis of aqueous solutions with and without a saline sample matrix. These studies focus on measurements at high pressure^4,14–20^, as well as at atmospheric pressure^21–27^ conditions. The underwater LIBS technique relies on focusing a high peak power pulsed laser into a liquid sample to generate a plasma. High temperature and pressure cause plasma expansion at supersonic velocities, creating a shockwave and cavitation bubble^28–31^. Cooling and decay of the plasma occurs by losing energy to the shockwave, spectral emission, and to the surrounding liquid.

This paper describes a laboratory prototype of an all optical down hole LIBS sensor for assessing groundwater quality^32–34^. The basic optical design of the prototype sensor head has been described, which includes an evaluation of the monolithic, passively Q-switched (PQSW) microchip (MC) laser. The fabricated sensor has been evaluated for its performance including determination of the Limit of Detection (LOD) of various metal species dissolved in water. While most LIBS systems utilize an actively Q-switched laser, use of a PQSW laser simplifies the design of downhole components and increases sensor robustness. Development of a low-cost fiber coupled sensor head allows the expensive and fragile pump laser and spectrograph to remain on the surface in a safe and controlled environment while only the sensor head needs to enter a potentially hostile environment.

## Materials and Methods

A quasi-CW 808 nm fiber coupled diode laser (Apollo Instruments, F700-808-6) was used for pumping the sensor head. The pump laser produced 600 μs square pulses with an energy of 200 mJ at a repetition rate of 5 Hz driven by a Northrop Grumman eDrive diode driver. The pulse energy measurements were made with a pyroelectric meter (Ophir, PE25BF-C) and the temporal beam profiles were measured with a biased silicon photodiode (ThorLabs, DET10A) and a 500 MHz oscilloscope (Tektronix, TDS3052). Neutral density filters were used to reduce the incident laser intensity on the photodiode to prevent saturation and ringing. The beam profile and quality were determined using a Spiricon M2-200 optical train equipped with a 500 mm focal length lens and a CCD camera (ThorLabs, DCC1545M). The spectra were recorded by using a fiber coupled Czerny-Turner spectrograph (Andor, Shamrock 303i) and a gated TEC cooled ICCD camera (Andor, iStar DH320T-25F-03). The ultrafast gate setting of the iStar ICCD camera was used, giving a trigger insertion delay of approximately 35 ns. An optical fiber and a coaxial cable of equal length were used to transfer the plasma emission to the spectrograph and the trigger to the camera, respectively. As the speed of the optical signal in the fiber optics and the electronic signal on the co-axial cable was approximately the same, no additional delay in signal was added. Calcium chloride dihydrate (Fisher, BP510), strontium chloride hexahydrate (Alfa Aesar, 3339322), potassium chloride (Alfa Aeser, 1159530), and deionized water (Millipore, Milli-Q (resistance 18 MΩ)) were used to prepare aqueous solutions. During the measurements, the solutions were contained in a 1 cm quartz cuvette at room temperature and atmospheric pressure.

## Results and Discussion

### Optical design

**Figure 1:**
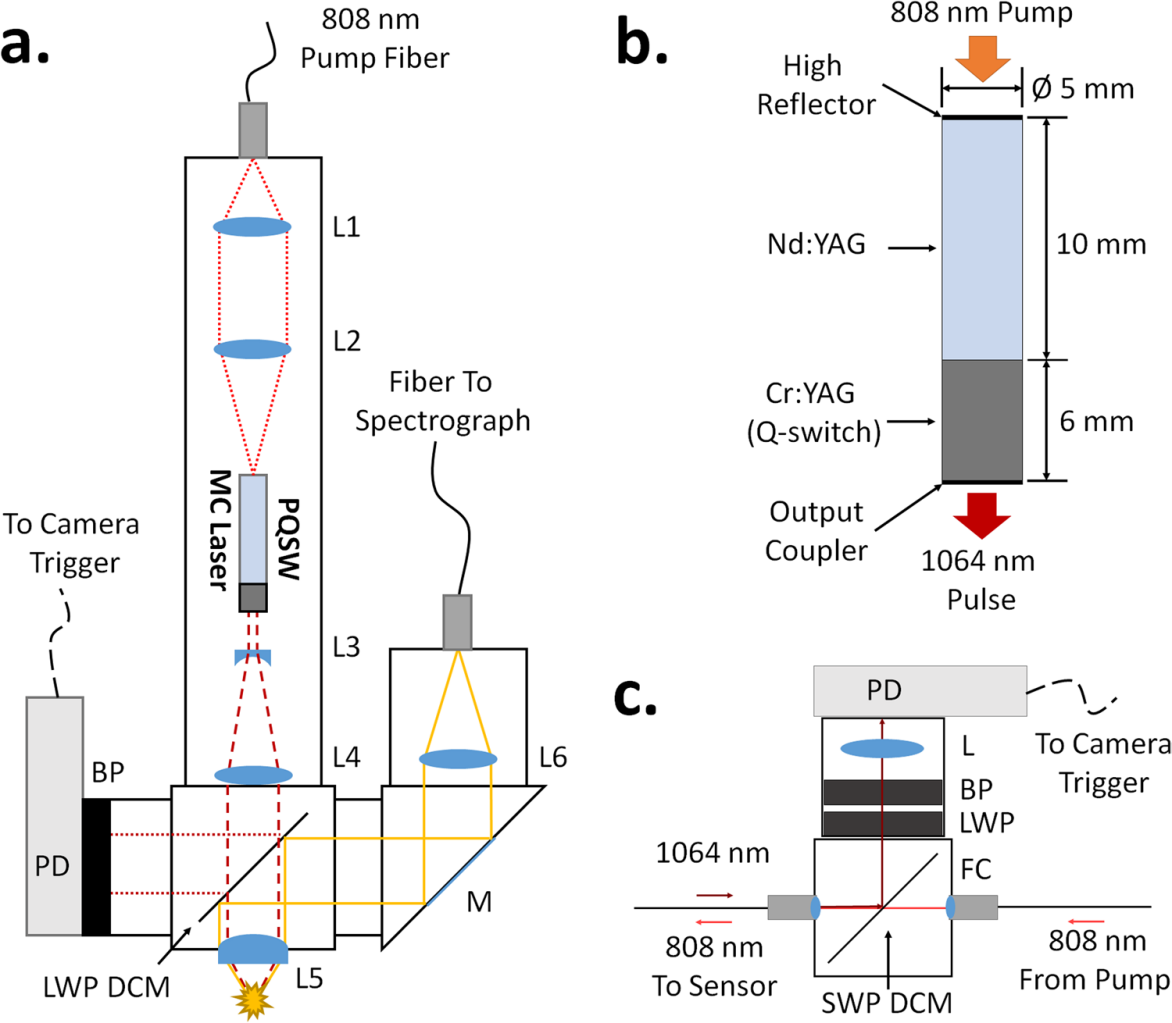
Optical design of sensor and components. (**a**) Schematic layout of LIBS sensor head. Pump coupling lenses L1 = 25 mm and L2 = 50 mm, Beam expander L3 = −25 mm and L4 = 75 mm, Aspheric focusing lens L5 = 10 mm, Emission coupling lenses L5 and L6 = 50 mm. LWP DCM = 900 nm LWP dichroic mirror, M = aluminum mirror, BP = 1064 nm bandpass filter, PD = photodiode. (**b**) Design of the monolithic PQSW Nd:YAG microchip (MC) laser. (**c**) Alternative detector triggering scheme that places the PD in line with the pump fiber. BP = 1064 nm bandpass filter, LWP = 900 nm LWP filter, L = 20 mm lens, SWP DCM = 900 nm SWP dichroic mirror, FC = fiber collimator (one of two).

The prototype sensor head, shown schematically in Fig. 1a, is housed within a 30 mm cage system (ThorLabs). The pump laser beam is collimated and focused into the PQSW MC laser using anti-reflective (AR) coated spherical lenses (L1 and L2). The MC laser output is then expanded by a 3X beam expander (L3 and L4) and passes through a 900 nm long wave pass (LWP) dichroic mirror (ThorLabs, DMLP900R). The resulting beam is finally focused by a near infrared (NIR) AR coated aspheric lens (L5) with a numerical aperture (NA) of 0.55 (ThorLabs, AL1210-C). Light emitted by the laser induced plasma (LIP) is collected in a confocal manner by the same aspheric lens and redirected toward a fiber connecting to the spectrograph via the LWP dichroic mirror (DCM). The high NA and aspheric shape of this lens aids in signal collection which is important for producing a sensitive LIBS probe. Triggering for the camera is supplied by a biased photodiode equipped with a 1064 nm bandpass filter, which monitors the back-surface reflection from the dichroic mirror. An alternative triggering scheme useful for downhole measurements is discussed in a later section.

A major advantage of this confocal design is that the alignment of the optics for the plasma emission and the laser excitation pulse is greatly simplified because both the LIP emission and excitation pulse share the same focal point. However, it is necessary that the focusing optics be highly transmitting in both the infrared and visible range to maximize the collected signal and delivered excitation laser and to avoid possible damage from the high peak power of the laser. Due to a lack of a commercially available lens with such a coating, a choice was therefore made to use a NIR AR coated lens to avoid damage from the pulsed laser. According to the manufacturer this coating is designed for use in 1050–1700 nm range, however, we could obtain acceptable transmission outside this range. It is expected that the use of a custom coating could further improve the optical properties for use in both the visible and UV range.

**Figure 2:**
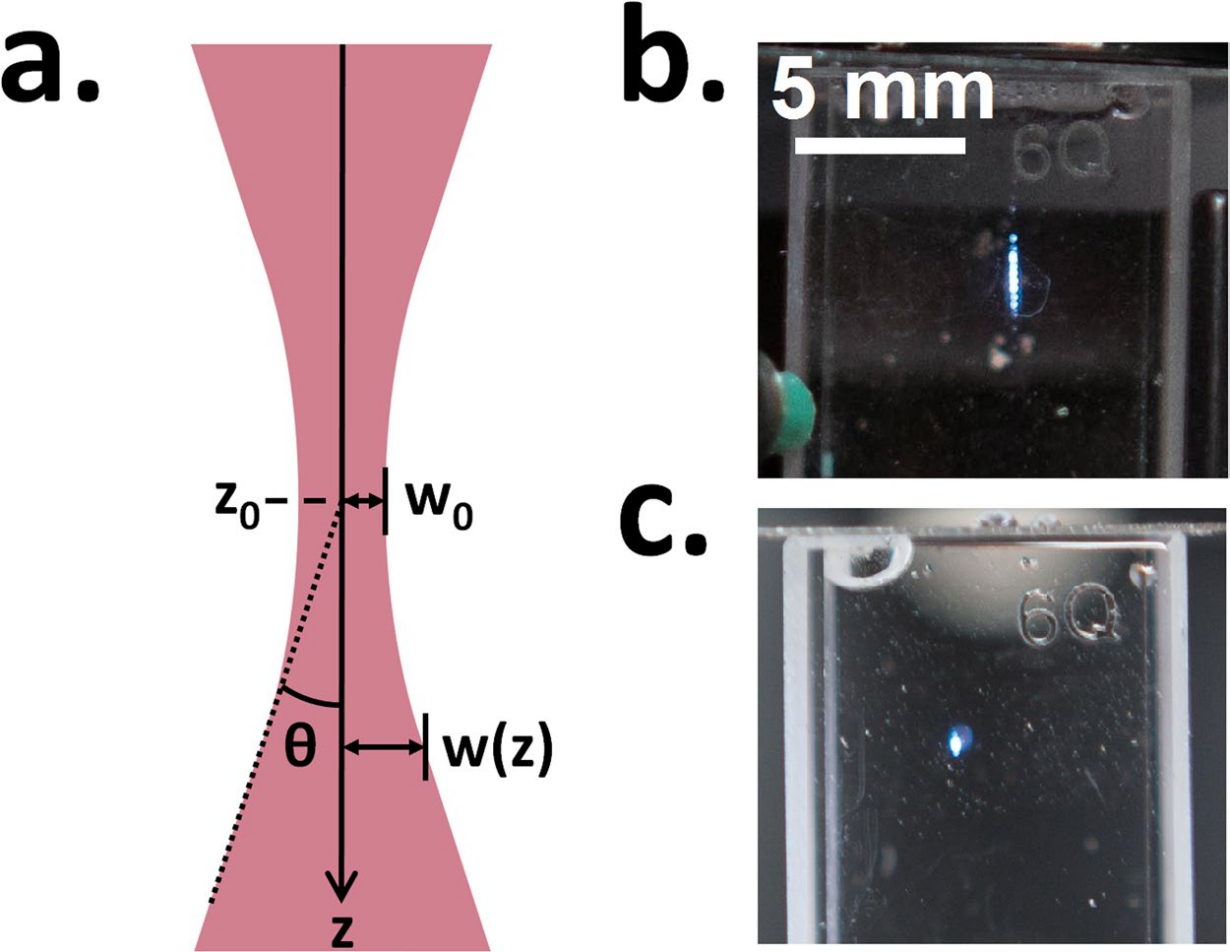
Properties of focused beam. (**a**) Beam radius, ‘w(z)’, as a function of position along propagation direction, ‘z’, where z_0_ = beam focal point, w_0_ = beam waist radius, and θ = beam divergence half angle. (**b**,**c**) - Effect of 3X beam expander (L3 and L4, Fig. 1) on laser induced plasma (LIP) in water. Without (**b**) and with (**c**) beam expander. Note, the laser beam is entering from the top of the cuvette in these images.

During early testing it was noticed that the prototype produced an elongated LIP in water (Fig. 2b) and was unable to cause breakdown in air due to low laser power density (irradiance) at the focal point. Therefore, a decision was made to add a beam expander (L3 and L4, Fig. 1a) in anticipation that it would increase the power density at the focal point and result in a brighter, more compact spark (Fig. 2c). Figure 2a shows the evolution of the laser beam radius, ‘w’, along the propagation direction in the vicinity of a focal point. Note that the beam radius reaches a finite minimum radius, w_0_, this point is known as the beam waist.

**Figure 3:**
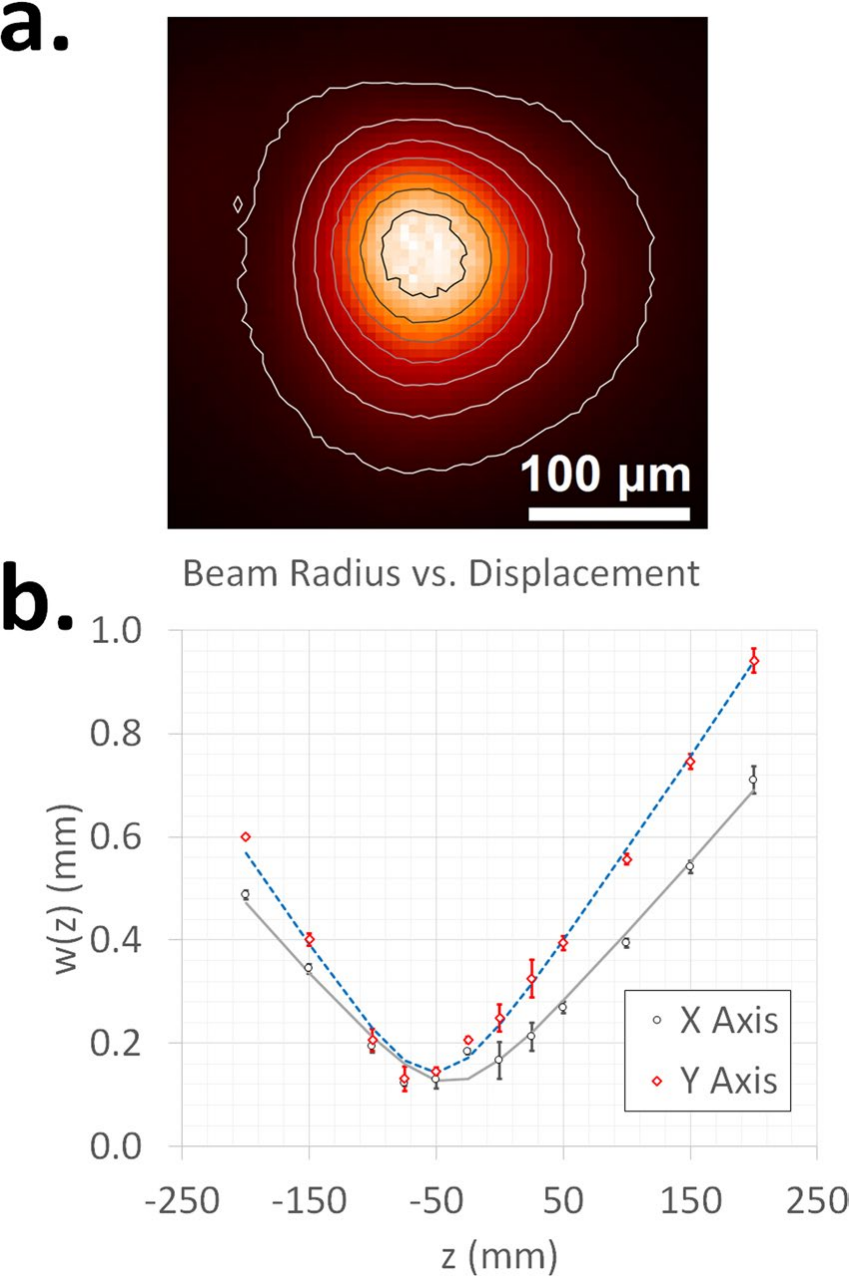
(**a**) Beam intensity profile (near the focal point of a 500 mm lens). (**b**) Measured beam radius as a function of displacement along beam propagation direction. Error bars are ±three standard deviations of six measurements at each point. The intensity profile was plotted using MatPlotLib^47^.

Since the focal point irradiance is inversely proportional to the focused beam cross-sectional area, reducing the beam waist radius (w_0_) will increase the power density by the same factor squared. The theoretical beam waist radius for a Gaussian beam depends on the incoming beam radius as well as the lens focal length and can be calculated by using Eq. 1.

1 $${w}_{0}={M}^{2}\frac{\lambda }{\pi }\frac{f}{R}$$

where w_0_ = beam waist radius (beam radius is defined here as the distance to 2σ or 1/e^2^ the peak intensity), λ = wavelength, f = lens focal length, and R = beam radius at the focusing lens. For an ideal Gaussian beam, M^2^ = 1, while for a beam that deviates from an ideal Gaussian, w_0_ increases by a factor M^2^ > 1.

According to Eq. 1, expanding the beam radius (R) before the focusing lens (L5, Fig. 1a) is expected to reduce w_0_ by the same factor. Therefore, a 3X Galilean beam expander was chosen as it would result in a significant increase in power density (9X) while not adding excessive length to the design (+50 mm for the selected lenses). This resulted in a brighter and more compact plasma in water (Fig. 2c) and enabled the prototype to produce a LIP in air [requiring a power density >10^11^ W/cm^2^ ^35^]. While reflection losses from the additional beam expander optics will reduce the available pulse energy (even with an AR coating), the gain in energy density realized from the reduced beam waist radius outweighs the reflection losses.

### Laser Pumping

The sensor head described in this paper utilizes a diode-pumped PQSW solid state laser. Use of a diode pumped PQSW laser has several advantages: (1) Diode pumping simplifies maintenance by eliminating the need for lamps which typically last up to a few thousand hours while the run time for diode lasers can be on the order of 100,000 hours, (2) the low peak power of the quasi-CW pump laser can be easily coupled into and transmitted by optical fibers without risk of damage, removing the need for close proximity of the pump source and solid-state laser, and (3) a PQSW laser eliminates the need for active switching elements such as a Pockels cell or an acousto-optic modulator (AOM) within the sensor head. By eliminating the lamps and active switches there is no need for high voltage or high current to be transmitted to the sensor head nor a need for fragile and temperature sensitive components to be in the sensor head. Furthermore, the ability to generate the high peak power pulse within the sensor head means this pulse does not have to be coupled into and transmitted by the fiber optics, significantly reducing the risk of fiber damage^36^.

### Detector Triggering

The triggering device, attached to the sensor head, is shown in Fig. 1a. As indicated in section 3.1, triggering is achieved by monitoring the back-surface reflection of the 1064 nm pulse from the 900 nm LWP DCM using a photodiode (PD). Although the LWP DCM is designed to transmit all the 1064 nm emission, a part of the emission is still reflected by the back surface. This back-surface reflection is strong enough to be detected by the photodiode even without focusing optics. A band pass filter is included to remove the residual 808 nm pump light that had not been absorbed by the laser crystal and could saturate the photodiode. By triggering the camera from the MC laser emission instead of the pump laser, any issues resulting from timing jitter between the emission of the 808 nm pump and emission of the 1064 nm pulse are avoided.

The current triggering device, described above and shown in Fig. 1a, is designed for use in the laboratory setting. However, if the LIBS sensor were to be deployed for *in situ* measurements in a downhole environment, an all optical design is preferred to eliminate the need for sub-surface electronics and signal or power cables to be run downhole. Thus, a modified triggering device was designed and tested (Fig. 1c). In this scheme the triggering device can be placed in line with the pump fiber for detection of a small percentage of the 1064 nm laser pulse that leaks through the MC laser’s high reflector and travels back up the pump fiber. Upon the backward travelling 1064 nm pulse reaching the triggering device, a 900 nm short wave pass (SWP) DCM redirects it toward a photodiode (PD) for detection while the 808 nm pump light passes through the device unimpeded (Fig. 1c). This design allows the triggering device to be placed anywhere along the pump fiber length. By placing the device on the surface near the pump laser and spectrometer it will be kept away from the harsh downhole environment. It should be noted that the 1064 nm pulse traveling backwards up the pump fiber is significantly lower in intensity as compared to the LIP excitation pulse, therefore, a lens (L, Fig. 1c) was used to focus the pulse onto the diode to enable its detection. Measurements confirmed that the resulting pulse is intense enough to be easily detected on an oscilloscope. In fact, within the sensor head the backward traveling pulse will be coupled into the pump fiber at approximately same time the LIP is created. However, the delay time between the arrival of the plasma emission and backward traveling laser pulse to the spectrograph and triggering device can be adjusted by appropriate choice of the difference in length between the two optical fibers, allowing for triggering to be activated at the desired time.

It must be noted that due to a large amount of reflected 808 nm pump light, two stages of filtering (1064 nm bandpass and 900 nm LWP) was necessary to prevent saturation of the detector. Testing of this triggering device (Fig. 1c) showed a 12% loss of pump power. It is likely that most of this pump loss can be avoided by using an 808 nm AR coated fiber as is there a ~4% reflection loss from each end of the uncoated fiber.

### Laser Design

The design of the MC laser is shown in Fig. 1b. It is a monolithic plane-parallel cavity consisting of a 10 mm Nd:YAG crystal (0.9% Nd^3+^) diffusion bonded to a 6 mm Cr:YAG passive Q-switch with an initial 1064 nm transmission of 30% (Photop Technologies). The high reflector is coated directly onto the Nd:YAG face and the output coupler (30% reflectivity) is coated directly onto the Cr:YAG face. This configuration ensures the cavity alignment needed for optimum performance of the MC laser.

### Laser Performance

#### Pulse Energy and Repetition Rate

The repetition rate and pulse energy of the MC laser depend on the pump rate and pumped volume of the laser crystal respectively. Please note that the pump rate is defined as the pump energy delivered to the laser crystal per unit volume per unit time and the pumped volume is defined as the volume of the laser crystal excited by the pump. For a PQSW laser, a higher pump rate generally results in a higher pulse repetition rate and a larger pumped volume yields a higher pulse energy. In this study, the pump rate was varied by changing the position of lens L2 (Fig. 1a) while the pump energy and the pump pulse length were kept fixed. It should be pointed out that in the method used here the pump rate and pumped volume of the MC laser were not independent, which is generally not a requirement for laser operation. In this case, the maximum pump rate was achieved by tightly focusing the pump into the Nd:YAG crystal, which resulted in the emission of multiple nanosecond pulses of 2.4 mJ each per pump pulse. It was therefore, decided to defocus the pump, to reduce the pump rate and ensure the generation of a single nanosecond laser pulse of 3 mJ per pump pulse. Single pulse operation with an average pulse energy of 3.0–3.1 mJ was used throughout this study.

#### Beam Profile

The beam radius was determined by performing a knife-edge measurement^37^ and was found to be 0.37 mm within 5 mm of the output coupler. It should be noted that the radius in this study is defined as the distance from the center of the beam where the intensity drops to 1/e^2^ (approximately 13.5%) of the maximum intensity. The beam divergence was measured by determining the beam radius at two points separated by 11.5 cm and was found to be 0.07°.

The M^2^ value represents the deviation of the beam cross section (i.e. the intensity profile) of a real laser beam from an ideal Gaussian beam and it is always greater than one. However, if this value is close to one the laser beam is considered to be of high quality. M^2^ can be calculated using Eq. 2 ^38^.

2 $${M}^{2}=\frac{\pi {w}_{0}\theta }{\lambda }$$

where w_0_ is the beam waist radius, θ is the beam divergence, and λ is the wavelength.

To determine the values of w_0_ and θ (Fig. 2a), the following relation (Eq. 3) can be used^38^.

3 $$w(z)=\sqrt{{{w}_{0}}^{2}+{\theta }^{2}{(z-{z}_{0})}^{2}}$$

where z_0_ is the focal point (the ‘z’ location of w_0_).

To compute M^2^, the beam radius as a function of ‘z’ was determined using the M2-200 system by measuring the intensity profile (see Fig. 3a for an example) at 12 points around the beam waist. The radius (‘w’) at each point was determined by fitting a two-dimensional Gaussian to the measured intensity profile^39^. Subsequently, the parameters w_0_, z_0_, and θ were determined by fitting Eq. 3 to the measured w(z) (Fig. 3b). Once w_0_ and θ were known, M^2^ can be calculated using Eq. 2. This measurement was repeated six times and the calculated average values of M^2^, w_0_, and θ are presented in Table 1 (uncertainties are +/− the standard deviation of the six measurements).

**Table 1:** Determined values of M^2^, w_0_, and θ from the data presented in Fig. 3b.

Axis	M^2^	w_0_ (μm)	θ (deg)
X	1.04 ± 0.02	125 ± 4	0.16 ± 0.003
Y	1.55 ± 0.03	142 ± 3	0.21 ± 0.002

Note that the values of w_0_ and θ are dependent on the focal length of the lens (500 mm) used in the M2-200 system.

The M^2^ value along with the beam radius can be utilized to estimate the beam waist radius for a given lens (Eq. 1). To calculate w_0_ for lens L5 (Fig. 1a) the M^2^ value of 1.55 (Table 1) and the beam diameter at L5 was used. A knife edge measurement was utilized to estimate the beam radius at the position of lens L5 (Fig. 1a), which was found to be 1.23 mm (after the 3X expander). By using the values of the beam radius (1.23 mm) and M^2^ (1.55), the focal length of L5, and the laser wavelength, w_0_ for L5 was calculated to be 4.3 μm (in air). Please note that w_0_ is expected to be larger in water due to its higher index of refraction.

#### Temporal Characteristics

**Figure 4:**
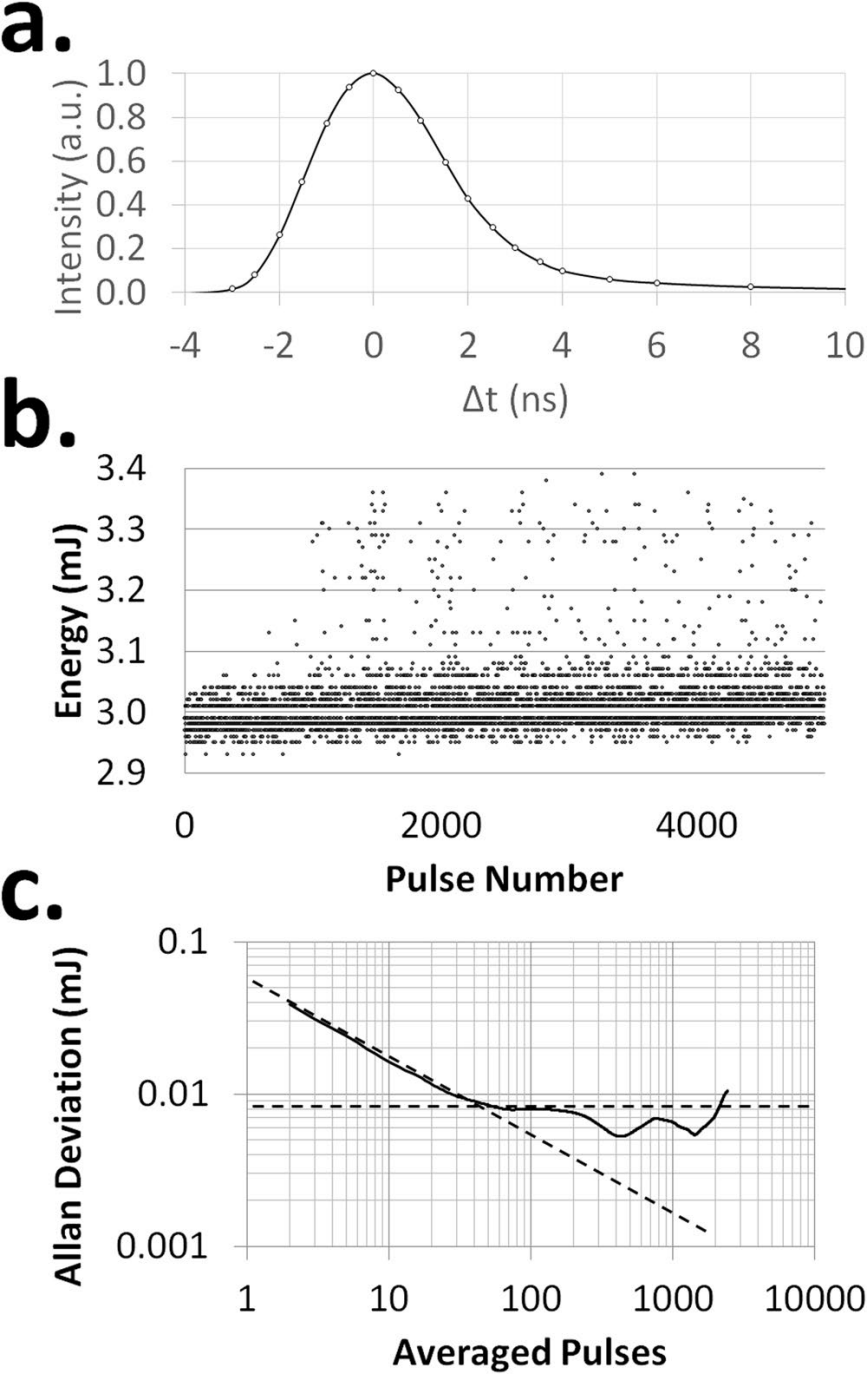
Temporal properties of laser. (**a**) Temporal profile of the MC laser pulse. Pulse width is 3.3 ns (FWHM) with a 1.8 ns rise time and 3.4 ns fall time. (**b**) The energy of five thousand laser shots as a function of shot number (pulse rate = 5 Hz). (**c**) The Allan Deviation showing two minima at ~450 and ~1500 shots. The dashed lines represent the contribution of white noise (downward sloping) and 1/f noise (horizontal).

The temporal profile of the MC laser pulse was measured with a photodiode as described in the material and methods section and the pulse width was determined to be 3.3 ns (FWHM, Fig. 4a). As can be seen in Fig. 4a the laser pulse is asymmetric with a 1.8 ns rise time (10–90% max intensity) and a 3.4 ns fall time (90–10% max intensity).

Laser characteristics such as beam divergence, M^2^, pulse length, and average pulse energy are important values for evaluating the performance of a laser and to determine if a given laser system can produce a LIP suitable for LIBS. Quantitative LIBS analysis relies on the intensity of the plasma emission to determine the elemental concentrations, and fluctuations of the pulse energy (i.e. laser intensity noise) add noise to the measured spectral intensity. Therefore, averaging data over many laser shots is a way of reducing the effect of these fluctuations. To determine the number shots to be averaged to achieve a desired precision requires an analysis of the intensity noise.

To quantify the MC laser’s intensity noise, the energy of 5000 pulses (Fig. 4b) was measured, giving an average pulse energy of 3.01 mJ and a standard deviation of 0.06 mJ or 2%. By using the relationship between the standard deviation (σ) and standard error of the mean (σ_mean_ = σ/√N, where N = number of measurements averaged) it is possible to estimate the number of laser shots needed to achieve the desired precision. Unfortunately, the standard deviation does not capture all the variations that can occur. A better way to evaluate the effect of signal averaging is achieved by using the Allan deviation^40,41^. Although the Allan deviation is often associated with phase and frequency measurements, it is applicable to voltage and intensity data as well^39,40,42^. Generally, longer integration times result in an increase in the signal to noise ratio (S/N). However, this is not always true as slowly varying fluctuations in the output can add to the noise if the integration time is sufficiently long. This means there is an optimum integration length beyond that the S/N will worsen. Figure 4c shows the overlapping Allan deviation computed for a set of 5000 pulses using AllanTools^43^. From this figure the optimum integration time for the MC laser (pumped as described earlier) was determined to be ~450 pulses. This figure also indicates that white noise dominates for integrations between 1 to 30 pulses and the 1/f noise floor becomes prominent around 70–150 pulses^42,44^. The dips occurring after 200 pulses are possibly due to a periodic perturbation^42,44^ from the cycling of the pump laser chiller, room air-conditioning, and/or other periodic sources causing minima at 1.5 and 5 minutes (i.e. 450 and 1500 pulses at 5 Hz).

### Prototype testing

The prototype was evaluated for its performance by analyzing Ca, K, and Sr solutions. Aqueous solutions of these elements were prepared as described in the materials and methods section. The laser spark was located 4–5 mm into the bulk liquid. Directing the laser beam through the side of the cuvette avoided complications arising from bubbles accumulating at the top of the cuvette (if covered) or splashing caused by the submerged spark (if uncovered). Quartz phosphorescence centered around ~400 nm^45^ was observed mainly for long gate widths (>10 μs), possibly the result of UV excitation of the cuvette by the plasma. Each measurement utilized the sum of 450 laser shots and eight replicates were used to generate the elemental spectra. A gate delay of 250 ns for Ca and 300 ns for Sr and K were used with a 3 μs gate width for all elements and the laser pulse energy used was 3.1 mJ for all measurements.

**Figure 5:**
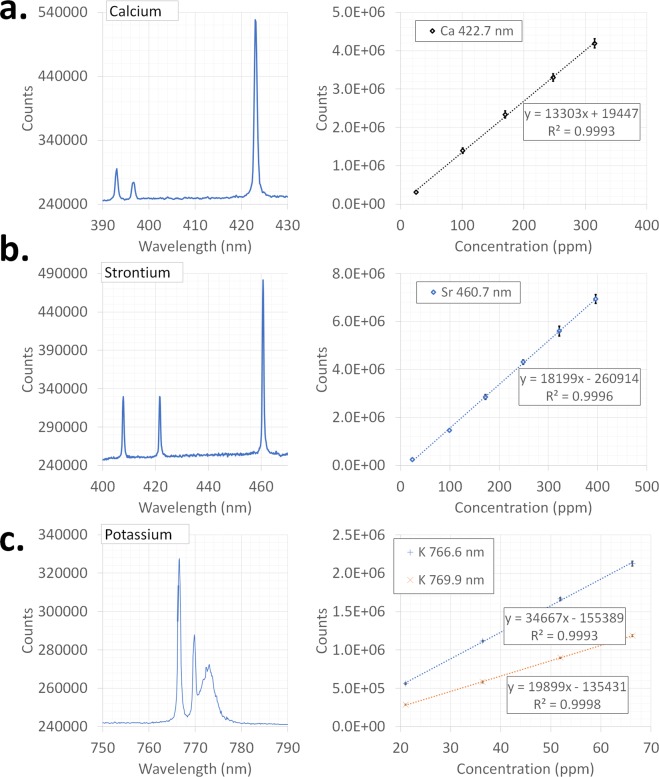
Emission spectra and calibration curves for the: (**a**) Calcium (422.7 nm line), (**b**) Strontium (460.7 nm line), and (**c**) Potassium (766.6 and 769.9 nm lines). The intensities for the calibration curves are the integrated intensities of the emission lines. Cation concentration for the emission spectra are 25.1 ppm Ca, 24.1 ppm Sr, 5.2 ppm K. Note the broad 773 nm peak interfering with the potassium spectrum. This peak was also present in DI water. Error bars are ±three standard deviations of eight measurements.

The emission spectra for Ca, K, and Sr are shown in Fig. 5. It should be pointed out here that the Ca 422.7 nm and Sr 460.7 nm peak intensities were extracted by fitting a single Lorentzian plus a linear background over the ranges 417–430 nm and 455–465 nm, respectively. Since the K spectrum includes two closely spaced analyte lines and an interfering line, these 3 peaks plus a linear background were fit simultaneously over the range 760–780 nm. While the K 766.6 and K 769.9 nm peaks were modeled as Lorentzians, the broad 773 nm peak was modeled as a Gaussian. However, for generating the calibration curves and calculating the LODs only the integral of the Lorentzian components was used. It should be pointed out here that the integrated intensity of the 773 nm peak was weakly dependent on the KCl concentration (rising ~30% as the KCl concentration increased from 21–52 ppm). Also, this peak is present both in the KCl sample and DI water and is likely due to a broadened oxygen line or a luminescent contaminant in the cell or optics that was excited by the plasma emission. The calibration curves for Ca, Sr and K with the corresponding spectral lines for each analyte are presented in Fig. 5. It should be noted that self-absorption of the ionic lines of Ca and Sr were observed over the concentration ranges used in this experiment. No self-absorption however, was observed with the measured atomic Ca, Sr, and K lines. Error bars in each case are three standard deviations (99% confidence interval) of eight measurements. The calibration curves for all the elements show a good linearity with R^2^ values over 0.99.

The limits of detection (LOD) for Ca, K, and Sr shown in Table 2 were estimated using Eq. 4.

4 $$LOD=3\sigma /m$$

where σ is the standard deviation (σ) of eight LIBS intensities (450 laser shots each) of deionized water at the peak location (e.g. 422.7 nm for Ca, etc) and m is the slope of the calibration curve (Fig. 5). The LODs obtained in this study are comparable to previously reported values obtained in ambient conditions (Table 2).

**Table 2:** Room temperature and pressure limits of detection for Ca, Sr, and K.

Element	Line (nm)	LOD (ppm)	LOD (literature) (ppm)		
Calcium	422.7	0.10^A^	0.94^B,†^	0.047^E^	0.13^G^
	393.4^‡^			0.01^F,Δ^	0.6^G^
Strontium	460.7	0.04^A^	2.89^B,†^		
	421.5^‡^		0.34 ^C,#^		
	407.8^‡^		0.025^D^		
Potassium	766.6	0.009^A^	0.03^B,†^	0.006^F,Δ^	1.2^H^
	769.9	0.069^A^			

^**A**^This study, ^**B**^Goueguel *et al*.^22^, ^**C**^Fichet *et al*.^24^, ^**D**^Popov *et al*.^26^, ^**E**^Pearman *et al*.^23^, ^**F**^Golik *et al*.^48^, ^**G**^Knopp *et al*.^25^, ^**H**^Cremers *et al*.^21^, ^**‡**^Lines showed self-absorption over the concentration ranges used in this study thus these lines were not used for calibration, ^**†**^NaCl solution matrix, ^#^LIP on liquid surface, ^**Δ**^fs LIBS + LIP on liquid surface.

The laboratory prototype described in this paper could easily be ruggedized for downhole measurements by enclosing it in a pressure resistant vessel. The fiber coupled design allows the sensitive components such as the pump laser and spectrograph to be separated from the sensor head so that only the compact, low cost head will be exposed to the adverse downhole conditions. Currently the prototype is approximately 35 cm in length with a 10 × 5 cm cross section at its widest point. Using off the shelf components, a miniaturized version of the described sensor head has been constructed that is almost half the length and 85% the maximum width of the original design^46^ while retaining the same performance. The current design will fit into a commercially available 6″ diameter pressure enclosure while the off the shelf miniaturized design can fit into a 4″ diameter commercial enclosure. A fully custom design could reduce the cross section significantly, which is limited primarily by the minimum distance between the mirrors (M and DCM in Fig. 1a) needed to redirect the LIP emission unobstructed and the size of the optical component themselves.

## Conclusion

A laboratory prototype of a downhole LIBS sensor based on a custom monolithic PQSW Nd:YAG laser and off the shelf optical components has been demonstrated. The prototype laser has excellent optical performance and sufficient energy to generate a LIP in both air and DI water at atmospheric pressure. Measured LODs for three elements relevant to geologic carbon sequestration are similar to or better than those obtained from actively Q-switched high power, bench top LIBS systems. This excellent performance is the result of the high laser beam quality, a compact spark, and the light gathering power of the signal collection optics. The developed prototype can potentially be used for other applications such as environmental monitoring, industrial process monitoring, and mineral resource characterization.

The datasets generated during and/or analyzed during the current study are available from the corresponding author on reasonable request.
